# Health accounts from past to present for a political arithmetic

**DOI:** 10.26633/RPSP.2021.73

**Published:** 2021-06-11

The *Pan American Journal of Public Health* informs the readers about involuntary errors in the following manuscript, as indicated by the authors:

In 2010, the World Health Assembly approved the recommendation of reporting health expenditures using standardized rules (3).

Should read as:

In 2011, the World Health Assembly approved the recommendation of reporting health expenditures using standardized rules (3)

Access to Health and Universal Health Coverage referred concretely to the need to “increase and improve investment in health,” specifically from public sources (4).

Should read as:

Access to Health and Universal Health Coverage referred concretely to the need to “increase and optimize public financing for health“ (4) or investment in health, specifically from public sources.

the WHO Asia and Pacific Regional Offices

Should read as:

WHO Southeast Asia and the Western Pacific Regional Offices

in the Strategy for Universal Access to Health and Universal Health coverage (4) as “the most important barrier to access and most inefficient source of funding.”

Should read as:

in the Strategy for Universal Access to Health and Universal Health coverage (4) as a barrier to access, and represents the most inefficient source of funding.

